# Dual Specificity Kinase DYRK3 Promotes Aggressiveness of Glioblastoma by Altering Mitochondrial Morphology and Function

**DOI:** 10.3390/ijms22062982

**Published:** 2021-03-15

**Authors:** Kyeongmin Kim, Sungmin Lee, Hyunkoo Kang, Eunguk Shin, Hae Yu Kim, HyeSook Youn, BuHyun Youn

**Affiliations:** 1 Department of Integrated Biological Science, Pusan National University, Busan 46241, Korea; minnnny@gmail.com (K.K.); smlee1048@gmail.com (S.L.); kanghk94@gmail.com (H.K.); egshin94@gmail.com (E.S.); 2 Department of Neurosurgery, Haeundae Paik Hospital, Inje University College of Medicine, Busan 48108, Korea; hykim0803@paik.ac.kr; 3 Department of Integrative Bioscience and Biotechnology, Sejong University, Seoul 05006, Korea; 4 Department of Biological Sciences, Pusan National University, Busan 46241, Korea

**Keywords:** DYRK3, glioblastoma multiforme, radioresistance, mitochondrial fission

## Abstract

Glioblastoma multiforme (GBM) is a malignant primary brain tumor with poor patient prognosis. Although the standard treatment of GBM is surgery followed by chemotherapy and radiotherapy, often a small portion of surviving tumor cells acquire therapeutic resistance and become more aggressive. Recently, altered kinase expression and activity have been shown to determine metabolic flux in tumor cells and metabolic reprogramming has emerged as a tumor progression regulatory mechanism. Here we investigated novel kinase-mediated metabolic alterations that lead to acquired GBM radioresistance and malignancy. We utilized transcriptomic analyses within a radioresistant GBM orthotopic xenograft mouse model that overexpresses the dual specificity tyrosine-phosphorylation-regulated kinase 3 (DYRK3). We find that within GBM cells, radiation exposure induces DYRK3 expression and DYRK3 regulates mammalian target of rapamycin complex 1 (mTORC1) activity through phosphorylation of proline-rich AKT1 substrate 1 (PRAS40). We also find that DYRK3 knockdown inhibits dynamin-related protein 1 (DRP1)-mediated mitochondrial fission, leading to increased oxidative phosphorylation (OXPHOS) and reduced glycolysis. Importantly, enforced DYRK3 downregulation following irradiation significantly impaired GBM cell migration and invasion. Collectively, we suggest DYRK3 suppression may be a novel strategy for preventing GBM malignancy through regulating mitochondrial metabolism.

## 1. Introduction

Glioblastoma multiforme (GBM) is a grade IV brain tumor according to the World Health Organization (WHO) classification and constitutes roughly 15% of all primary brain tumors. Unfortunately, GBM patients on average only survive 12–18 months after their initial diagnosis and only 5% of patients survive longer than five years. Due to its spatial selectivity, radiotherapy is one of the most effective therapies for eliminating residual GBM cells; however, tumor cells that survive radiotherapy acquire radioresistance, which is characterized by increased metastatic potential and proliferation [1]. Recently, cancer metabolism (characterized by a highly biased glycolytic activity, and also described as the Warburg effect) was shown to be a dominant regulator of therapeutic resistance [2,3]. Additionally, kinase expression and activity were shown to determine metabolic flux in tumor cells [4,5,6]. Previous studies have used transcriptomic and proteomic analyses of the kinome to characterize changes in kinase expression and phosphorylation state [7,8]. However, how kinases regulate GBM metabolism and therapeutic resistance is still largely unknown. In this study, we used microarray analyses of radioresistant GBMs, as well as the TCGA database, to characterize the kinome and investigate novel metabolic changes during acquired GBM therapeutic resistance.

Dual specificity tyrosine-phosphorylation-regulated kinase 3 (DYRK3) is a member of DYRK family that also includes DYRK1A, DYRK1B, DYRK2, and DYRK4. DYRK proteins catalyze autophosphorylation of serine/threonine and tyrosine residues [9]. While DYRK family members have similar structures and conserved catalytic domains, individual members have unique N-terminal and C-terminal regions that are responsible for their substrate specificity and unique functions in cancer cells. DYRK3 specifically has oncogenic or tumor-suppressive roles based on its phosphorylation target. One study suggested DYRK3 promotes cell survival by reducing p53 activity through the phosphorylation of SIRT1 [10]. Conversely, the low phosphorylation of steroid receptor coactivator protein 3 (SRC-3) by DYRK3 promoted purine metabolism and hepatocellular carcinoma progression [11]. Another study showed DYRK3-mediated direct phosphorylation of PRAS40 and mTORC1 activation, demonstrating the strong potential for DYRK3 in cancer signaling regulation [9]. The diverse roles that DYRK3 plays in tumor progression was also recently supported using data from a proteomic database, with targets of DYRK3 that included CREB and H3F3A [12]. Although a role for DYRK3 in brain tumors has not been reported, one previous study showed DYRK3 expression increased neuron dendrite growth, implicating DYRK3 involvement in nervous tissues [13].

Mitochondria are the main system of cellular respiration and are involved in numerous essential cellular functions, such as the regulation of energy metabolism, cell cycles, apoptosis, immune processes, oxidative stress, and calcium homeostasis [14]. Mitochondria continuously undergo dynamic processes, including fusion and fission, which are finely regulated and balanced in response to nutrient level [15]. Mitochondrial fusion mixes mitochondrial contents and prevents loss of important components, whereas mitochondrial fission splits mitochondrial contents during cell division and eliminates unhealthy mitochondria [16]. Recently, the balance of mitochondrial fusion and fission has been suggested as a key regulator of metabolic reprogramming, with tumor cells showing increased mitochondrial fission [17,18]. High levels of mitochondrial fission promote glycolysis and reduce oxidative phosphorylation (OXPHOS), while high mitochondrial fusion reduces glycolysis and increases OXPHOS. Therefore, it is critical to understand the regulation mechanism of mitochondrial dynamics. The GTPase dynamin-related protein 1 (DRP1) is a mediator of mitochondrial fission and has been widely studied due to its cytoplasmic localization and upregulation in cancer cells [18]. DRP1 is a target of many oncogenic kinases including AKT, MAPKs, and mTORC1, suggesting the importance of mitochondria dynamics during oncogenesis and tumor progression [19]. In GBM, high DRP1 expression is correlated with poor radiotherapy efficacy as well as increased mitochondrial fission, enhanced proliferation, invasion, and chemoresistance [20,21]. Further studies are required for a more precise understanding of the molecular mechanisms regulating mitochondrial dynamics in GBM.

In this study, we performed GBM-specific kinome analysis and identified DYRK3 as a factor capable of inducing GBM radioresistance. From our results and previous research [17,18,22], we hypothesize radiation-induced DYRK3 expression alters mitochondrial dynamics, leading to metabolic reprogramming and increased GBM aggressiveness. Given these functions of DYRK3, we suggest DYRK3 may be a therapeutic target for preventing GBM progression following radiotherapy.

## 2. Results

### 2.1. DYRK3 Is Upregulated by Radiation and Contributes to GBM Progression

**Figure 1:**
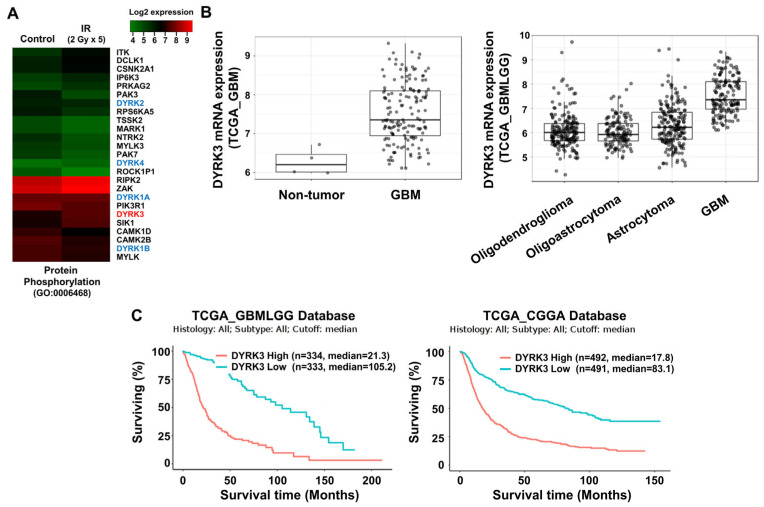

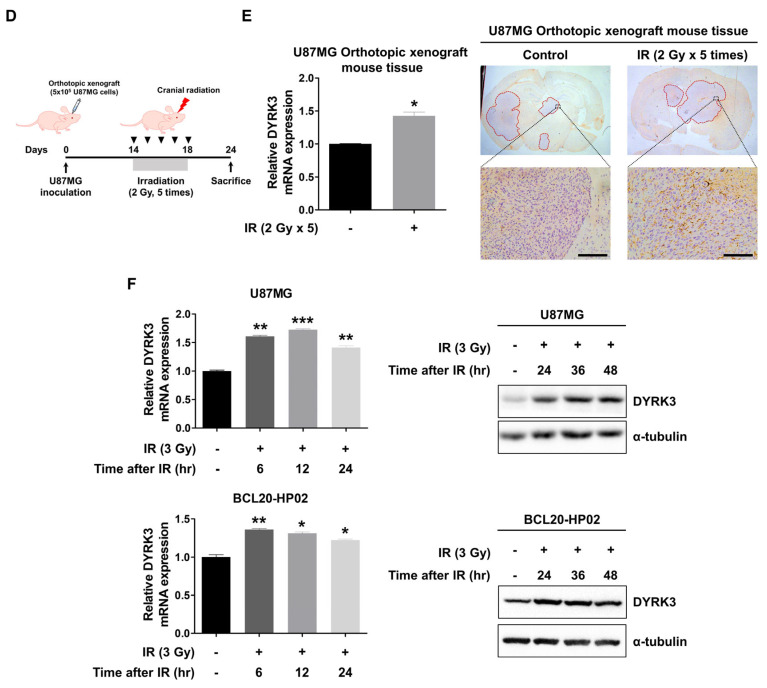
Dual specificity tyrosine-phosphorylation-regulated kinase 3 (DYRK3) is upregulated by radiation and contributes to glioblastoma multiforme (GBM) progression. (**A**) A heatmap of differentially expressed mRNA using the “protein phosphorylation” ontology term between control GBM samples and GBM samples exposed to irradiation. (**B**) Differential mRNA expression of DYRK3 in TCGA database. (**C**) The differential prognosis of glioma patients according to the mRNA expression of DYRK3. Data was obtained from GlioVis based on the TCGA database. (**D**) A summary of our orthotopic mouse model and irradiation schedule. (**E**) DYRK3 expression in GBM tissue of orthotopic mouse model with or without irradiation as analyzed by qRT-PCR (left panel) and IHC (right panel). Tumor borders are highlighted in red. * *p* < 0.05. Scale bars, 100 μm. (**F**) DYRK3 expression in GBM cells with or without irradiation as analyzed by qRT-PCR (left panel) and Western blot (right panel). * *p* < 0.05, ** *p* < 0.01, *** *p* < 0.001.

Although many kinases have been suggested as important factors controlling cancer malignancy, there have been few reports into the role of kinases to induce radioresistance in GBM [23]. To identify a kinase with a decisive effect on radioresistance of GBM cells, we conducted kinome analysis using two datasets. First, we screened genes with differential expression (>1.5-fold change) from a previous microarray profile (GEO accession number: GSE117126) and filtered out non-kinase genes (Figure 1A) [22].

Next, we used the TCGA database to determine correlations between differentially expressed genes and GBM patient prognosis. DYRK3 was selected for analysis due to its overexpression in irradiated GBM cells, its high expression in GBM patient tumors and its expression being associated with poor patient prognosis. We found that within the TCGA database DYRK3 mRNA levels are remarkedly high in GBM patients, as compared to both normal controls and other low-grade gliomas (Figure 1B). Furthermore, two glioma databases suggest patients with high DYRK3 expression have a median survival period that is roughly five times shorter than those with low DYRK3 expression (21.3 vs. 105.2 or 17.8 vs. 83.1 months, respectively. Figure 1C). To confirm increased expression of DYRK3 in GBM following radiotherapy, a GBM orthotopic xenograft mouse model was established using previous protocols (Figure 1D) [22]. U87MG cells, a GBM cell line, were intracranially injected into mice. After 2 weeks, mice were cranially irradiated (2 Gy/day for 5 serial days). Mice were then sacrificed one week after irradiation treatment and DYRK3 mRNA/protein expression was assessed. DYRK3 mRNA and protein levels were upregulated following irradiation, as shown by qRT-PCR and immunohistochemistry (IHC) (Figure 1E). In vitro experiments using two GBM cells (U87MG; GBM cell line, BCL20-HP02; patient-derived glioblastoma stem cell line) confirmed these in vivo findings, with irradiation increasing both DYRK3 mRNA and protein levels (Figure 1F). Taken together, we screened DYRK3 using kinome analysis of radioresistance and aggressiveness and experimentally confirmed elevated DYRK3 expression following irradiation.

### 2.2. Radiation-Induced DYRK3 Induces Mitochondrial Fission

**Figure 2:**
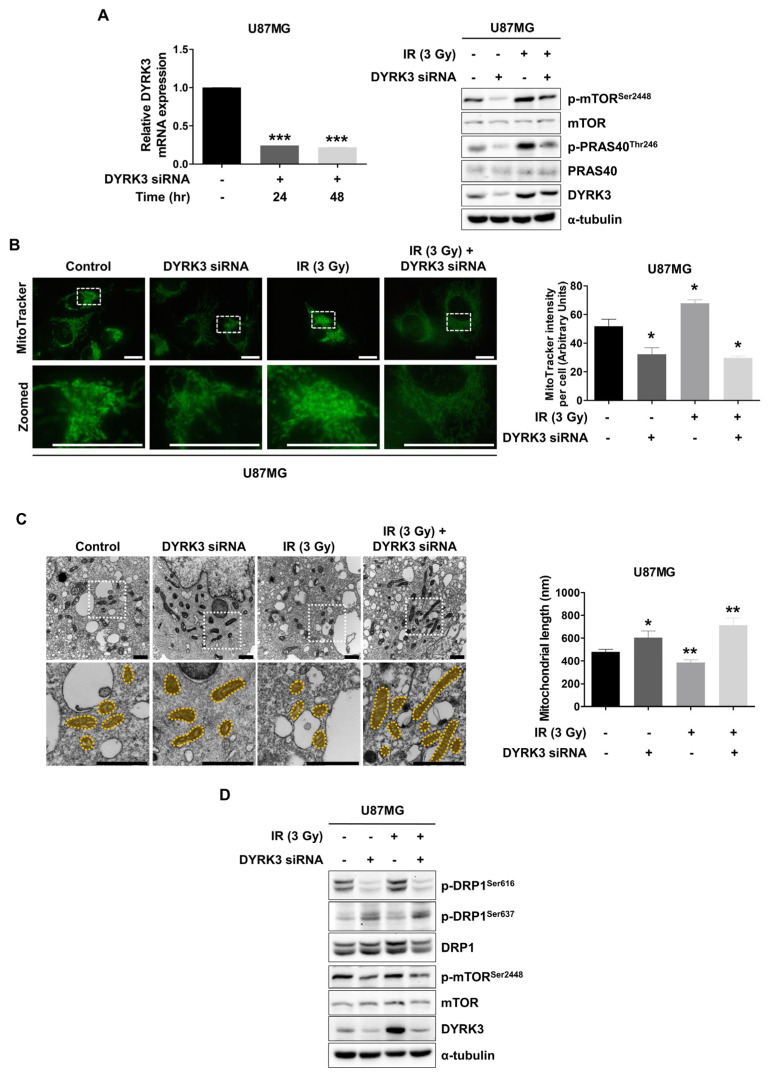
Radiation-induced DYRK3 induces mitochondrial fission. (**A**) Transfection efficiency of DYRK3 siRNA as assessed by qRT-PCR (left panel). *** *p* < 0.001. p-mTORC1 (ser2448), mTORC1, DYRK3, p-PRAS40 (Thr246), PRAS40, α-tubulin protein levels as detected by Western blot with or without knockdown of DYRK3 and irradiation (right panel). (**B**) U87MG cell mitochondrial mass with or without DYRK3 knockdown and irradiation as visualized by MitoTracker Green staining assay. Scale bars, 20μm. Quantification of MitoTracker intensity using ImageJ software (right panel). * *p* < 0.05. (**C**) Transmission electron microscopy (TEM) photomicrographs of U87MG cells with or without DYRK3 knockdown and irradiation (left panel). Quantification of mitochondrial length using ImageJ software (right panel). Mitochondria are highlighted in yellow. Scale bars, 1 μm. * *p* < 0.05, ** *p* < 0.01. Mitochondria number = 17. (**D**) p-DRP1 (Ser616), p-DRP1 (Ser637), Dynamin related protein 1 (DRP1), p-mTOR (Ser2448), mTOR, DYRK3, α-tubulin protein levels as detected by Western blot with or without DYRK3 knockdown and irradiation.

Although a few studies stated the signaling pathway in which DYRK3 is involved, a previous study suggested DYRK3 regulates mTORC1 signaling by directly phosphorylating PRAS40, a negative regulator of mTORC1 [9,24,25]. To characterize the DYRK3-PRAS40-mTORC1 signaling pathway in GBM cells, we performed Western blot assays following DYRK3 knockdown (Figure 2A). Consistently, phosphorylation of PRAS40 at Thr246 and mTOR at Ser2448 was reduced following DYRK3 knockdown, without changes to overall PRAS40 and mTOR levels. Conversely, irradiated U87MG cells showed elevated p-PRAS40, p-mTOR, and DYRK3 expression, an effect that was diminished when radiation was combined with DYRK3 knockdown. Previous studies have suggested activated mTORC1 signaling induces cancer metabolism through mitogenic gene expression synthesis; however, recent studies have also emphasized transitions in mitochondrial dynamics induced by mTORC1 activity [19]. 

Given that mTORC1 activation was altered by DYRK3, we performed microscopic analysis using MitoTracker Green FM to observe the mitochondrial morphology of U87MG cells following DYRK3 knockdown and irradiation (Figure 2B). Fluorescence images suggested mitochondrial mass is decreased in U87MG cells following DYRK3 knockdown. Interestingly, irradiation surviving cells displayed increased mitochondrial mass, which was reduced with DYRK3 knockdown. These results are consistent with the importance of mTORC1 in mitochondrial dynamics. It will be necessary to further examine precise mitochondrial morphology changes and how related regulators might affect morphology changes.

To further examine mitochondrial ultrastructural morphology, we used transmission electron microscopic (TEM) (Figure 2C). We found a significant increase in mitochondrial length in DYRK3 knockdown cells, as compared to control cells. Interestingly, we also found the mitochondria of irradiated DYRK3 knockdown cells were further elongated than that of DYRK3 knockdown alone cells, suggesting inhibiting radiation-induced DYRK3 expression deregulates mitochondrial fission in GBM cells. DRP1 is a key regulator of mitochondrial fission and its activity is regulated by two phosphorylation sites. Ser616 phosphorylation promotes mitochondrial fission, while Ser637 phosphorylation promotes mitochondrial fusion [26,27]. Following irradiation, we found p-DRP1 (Ser616) expression was increased, while p-DRP1 (Ser637) was decreased (Figure 2D). This effect was reversed following knockdown of DYRK3. Collectively, these results suggest radiation-induced DYRK3 expression promotes mitochondria fission, which is mediated by phosphorylation of p-DRP1 (Ser616). 

### 2.3. Radiation-Induced DYRK3 Inhibits OXPHOS and Increases Glycolytic Activity

**Figure 3:**
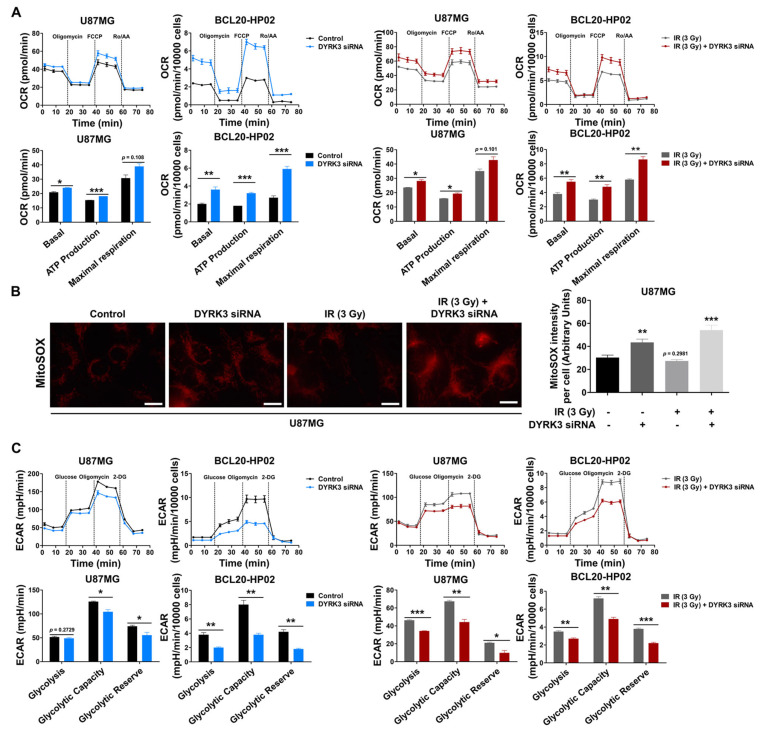
Radiation-induced DYRK3 inhibits OXPHOS and increases glycolytic activity. (**A**) Oxygen consumption rate (OCR) evaluation of electron transport chain (ETC) activity using a Seahorse XFp analyzer with sequential addition of 2.5 μM oligomycin, 1.5 μM FCCP, and 1.0 μM rotenone/antimycin A. * *p* < 0.05, *** *p* < 0.001. (**B**) U87MG cell mitochondrial superoxide with or without knockdown of DYRK3 and irradiation as visualized by MitoSOX red staining assay. Quantification of MitoSOX intensity using ImageJ software (right panel). Scale bars, 20μm. ** *p* < 0.01, *** *p* < 0.001. (**C**) Extracellular acidification rates (ECAR) evaluation of glycolytic flux using a Seahorse XFp analyzer. Glycolysis, glycolytic capacity, and glycolytic reserve were determined by the sequential addition of 10 μM glucose, 2.22 μM oligomycin, and 100 μM 2-DG. Values represent the mean ± SD of three experiments. * *p* < 0.05, ** *p* < 0.01, *** *p* < 0.001.

Mitochondria carry out cellular energetic metabolism and are regulated through a balance between mitochondrial fission and fusion. Given that mitochondrial fission reduces oxidative phosphorylation (OXPHOS) and reactive oxygen species (ROS) and increases glycolysis [17,18], we examined whether DYRK3 expression leads to these metabolic alterations. We used a Seahorse XFp analyzer to analyze oxygen consumption rate (OCR), as a representation of the electron transport chain (ETC), to determine the impact of DYRK3 on mitochondrial respiration. Respiratory capacity was determined by measuring OCR following the treatment of U87MG and BCL20-HP02 cells with oligomycin, FCCP, and rotenone/antimycin A. We observed a significant increase in cellular respiration in DYRK3 knockdown cells, as compared to control cells with or without irradiation (Figure 3A). As increased OXPHOS levels lead to increased ROS production, mitochondrial ROS levels were confirmed using MitoSOX Red staining. Fluorescence images showed mitochondrial superoxide was remarkably elevated upon DYRK3 knockdown with or without irradiation (Figure 3B). We next assessed extracellular acidification rates (ECAR) as a measure of glycolytic activity following glucose, oligomycin, and 2-deoxy-glucose (2-DG) treatments. ECAR was significantly reduced following DYRK3 knockdown (Figure 3C). Taken together, our results suggest DYRK3 maintains mitochondrial fission, favoring anaerobic glycolysis to avoid OXPHOS-generated ROS.

### 2.4. Radiation-Induced DYRK3 Promotes Migration and Invasion of GBM

**Figure 4:**
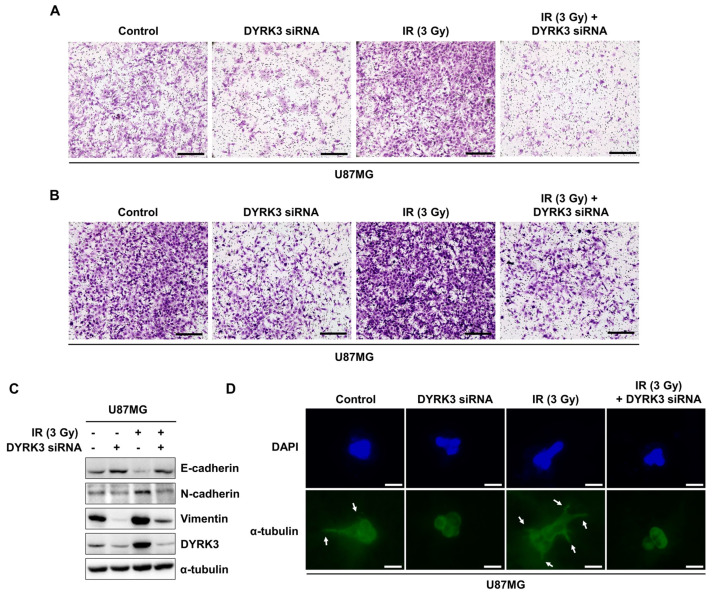
Radiation-induced DYRK3 promotes migration and invasion of GBM. (**A,B**) U87MG cell migration and invasion capacity after DYRK3 knockdown with or without irradiation as assessed by transwell migration or invasion assays. Scale bar, 100 μm. (**C**) U87MG cell Western blot analysis of migration capacity proteins after DYRK3 knockdown with or without irradiation. (**D**) Immunofluorescence staining with α-tubulin (green) and DAPI (blue) in a U87MG cell three-dimensional culture system. Arrows indicate changes in cell morphology to a spindle shape. Scale bar, 10 μm.

Previous studies indicate increased mitochondrial fission increases tumor cell metabolic advantages and lamellipodia formation. These changes promote the migration and invasion of various tumors, including GBM [28,29,30]. We investigated the effect of DYRK3 expression on cell migration and invasion using Boyden chamber assays. Migration assays measure cell migratory capacity towards a chemoattraction, while invasion assays measure both cell chemotactic and invasive capacity through extracellular matrices using matrigel. Irradiation markedly increased U87G cell migratory capacity, while DYRK3 knockdown inhibited U87G cell migration (Figure 4A). Increased inhibitory effect was observed with a combination of DYRK3 knockdown and irradiation, demonstrating the importance of DYRK3 suppression during irradiation. Similarly, irradiation increased invasive capacity and was reversed by DYRK3 knockdown with or without irradiation (Figure 4B). We next characterized the expression of epithelial-to-mesenchymal (EMT) markers (E-cadherin, N-cadherin, and Vimentin) in the context of irradiation and DYRK3 knockdown. Consistent with our migration and invasion assay results and independent of irradiation, E-cadherin (an epithelial marker) expression was increased following DYRK3 knockdown, while N-cadherin and Vimentin (mesenchymal markers) expression was reduced following DYRK3 knockdown (Figure 4C). Lastly, we used a 3D culture model capable of identifying morphologic alterations related to tumor growth. We found irradiated U87MG cells showed an increase in invasive features. However, these observed morphologic modifications were abrogated with DYRK3 knockdown (Figure 4D). Taken together, these results suggest DYRK3 knockdown can reverse many irradiation-induced GBM tumor cell migration and invasion features.

## 3. Discussion

**Figure 5:**
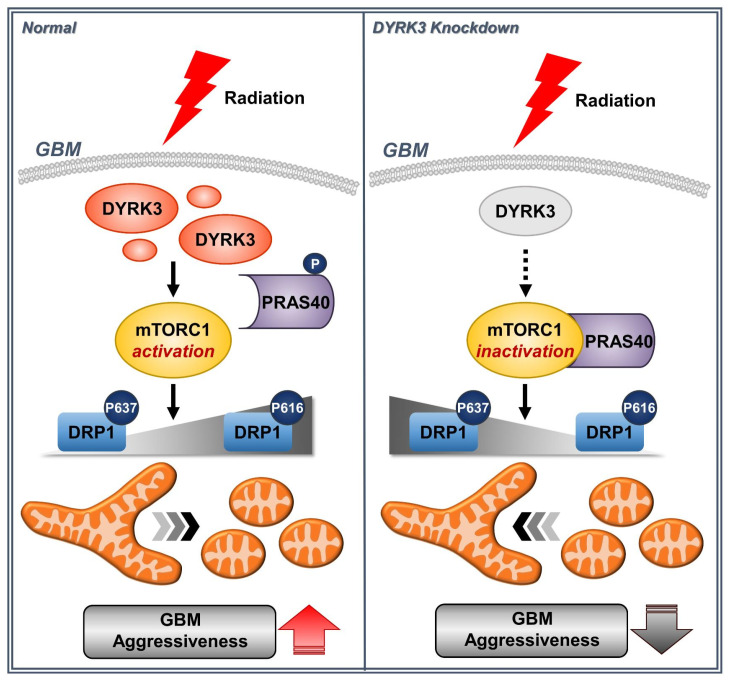
A model for how DYRK3 promotes GBM aggressiveness following irradiation. Radiation-induced DYRK3 expression increases mitochondrial fission through mTORC1 dependent DRP1 activation. Increased mitochondrial fission leads to increased aggressiveness of GBM.

GBM is a malignant brain tumor with poor prognosis and high therapeutic resistance. Recently, it has been suggested that metabolic reprogramming is significantly involved in therapeutic resistance. Furthermore, altered mitochondrial dynamics are suggested to control tumor cell metabolic flux [31,32]. In this study, we performed kinome analysis of microarray data from a radioresistant GBM orthotopic mouse model. We also used TCGA prognosis data to identify DYRK3 as a novel potential GBM target. As summarized in Figure 5, DYRK3 was increased following irradiation, promoting mTORC1 activity through PRAS40 phosphorylation and activating DRP1, a key regulator of mitochondrial fission. We also observed radiation-induced DYRK3 expression resulted in enhanced mitochondrial fission, glycolytic activity, and GBM cell invasiveness.

Altered DRP1-mediated mitochondrial dynamics are highly associated with cancer cell fate [18]. Previous studies determined that various oncogenic kinases drive DRP1 phosphorylation, suggesting DRP1 is an important mitochondrial dynamic checkpoint. ERK1/2 was reported to phosphorylate DRP1 at Ser616 for promoting mitochondrial fission and sustaining RAS-induced melanoma cell transformation and growth [33]. In nasopharyngeal carcinoma, AMP-activated protein kinase (AMPK), and cyclin-dependent kinase 1 (CDK1) axis activity determines the balance of DRP1 phosphorylation at Ser616 and Ser637, leading to chemoresistance [34]. CDK5 also phosphorylated DRP1 at Ser616 to increase mitochondrial fission in brain tumor initiating cells (BTIC), while Ca^2+^ calmodulin-dependent protein kinase 2 (CAMK2) phosphorylated DRP1 at Ser637, inducing mitochondrial fusion in non-BTIC tumor. These results significantly implicate mitochondrial dynamics in tumor differentiation and provide a basis for using kinase profiles to determine tumor cell mitochondrial dynamics [35]. Lastly, this previous study also suggested DYRK3 may be an upstream regulator of DRP1 activity and that DYRK3 modulation might alter mitochondrial dynamics in GBM. 

While DYRK family member functions have been reported in other tumors, to our knowledge this the first report of DYRK3 involvement in brain tumor progression. We note that we did not observe DYRK3 involvement in regulating cell proliferation, which is consistent with previous reports for DYRK family member functions. DYRK1A is associated with oncogenic protein inactivation and cell cycling in acute myeloid leukemia (AML) [36,37,38]. Conversely, DYRK1A and DYRK1B increases chemoresistance in gastric, ovarian, breast, and lung cancers [39,40,41]. In breast and ovarian cancer in particular, DYRK2 increases migratory activity of tumor cells through activation of c-JUN, c-Myc, and Snail signaling [42,43,44]. DYRK3 is reported to inhibit hepatocellular carcinoma progression through metabolic reprogramming mechanisms [11]. Little research exists on the functions of DYRK4; however, overexpression of the DYRK4-RAD51AP1 complex induces a more aggressive form of luminal breast cancer [45]. Collectively, DYRK family members increase tumor cell aggressiveness but not tumor proliferation and tumorigenesis. Microarray analysis from a radioresistant GBM mouse model (GSE117126) showed DYRK1A, DYRK1B, DYRK2, and DYRK4 expression does not significantly change (more than 1.5-fold upon) following irradiation. Compellingly, DYRK3 is the only DYRK family member that is correlated with glioma grade in the TCGA database. Given these findings, DYRK3 appears to mediate the majority of DYRK functions in GBM. Further studies are required to better understand this critical role of DYRK3 in GBM.

Previous studies have suggested inhibition of mitochondrial fission may be a therapeutic opportunity in cancer. Mitochondrial division inhibitor-1 (Mdivi-1) is the most well-known mitochondrial fission inhibitor, inhibiting DRP1 assembly and GTPase activity [46]. Dynasore inhibits mitochondrial fission by noncompetitively inhibiting DRP1 GTPase activity and P110 blocks DRP1 protein interaction and function [47]. While Mdivi-1, Dynasore, and P110 suppress tumor malignancy, direct inhibition of mitochondrial fission may be toxic to life. DYRK3 may be an upstream regulator of DRP1 and may be a target of chemical inhibitors such as Harmine and GSK-626616, which were proposed as novel and promising cancer treatment strategies [48,49]. Harmine in particular has been used in clinical studies and can penetrate the blood brain barrier, allowing for brain-specific delivery [50]. Follow-up studies will confirm the effect of these inhibitors in GBM patients with or without radiotherapy.

As GBM is currently one of the most malignant tumors, novel therapeutic strategies are required. We hope that our work describing the molecular relationship of DYRK3 within mitochondrial dynamics, and metabolic reprogramming will help clarify how DYRK3 alters GBM radioresistance and guide novel DYRK3-informed treatments for GBM patients.

## 4. Materials and Methods

### 4.1. Antibodies and Reagents

Primary antibodies specific for p-PRAS40 (Thr246), DRP1, p-DRP1 (Ser616), and p-DRP1 (Ser637) were purchased from Cell Signaling Technology (Cell Signaling Technology, Beverly, MA, USA) and specific for mTOR, PRAS40, E-cad, N-cad, and Vimentin were purchased from Santa Cruz Biotechnology (Santa Cruz Biotechnology, Santa Cruz, CA, USA) and specific for α-tubulin and p-mTOR (Ser2448) were purchased from Abcam (Abcam, Cambridge, MA, USA) and specific for DYRK3 was purchased from Thermo Fisher Scientific (Thermo Fisher Scientific, Cleveland, OH, USA). Secondary antibodies specific for mouse IgG and rabbit IgG were purchased from Enzo Life Sciences (Enzo Life Sciences, Ann Arbor, MI, USA). Eagle’s Minimum Essential Medium (MEM), Hanks’ Balanced Salt solution (HBSS), Phosphate Buffered Saline (PBS), and fetal bovine serum (FBS) were acquired from WelGENE Inc (WelGENE Inc., Daegu, Korea). Penicillin, streptomycin, recombinant human EGF protein, Trizol, MitoTracker^TM^ Green FM and MitoSOX^TM^ Red were obtained from Thermo Fisher Scientific (Thermo Fisher Scientific, Cleveland, OH, USA). SiRNAs specific for human DYRK3 and control siRNA were purchased from Bioneer (Bioneer, Daejeon, Korea).

### 4.2. Cell Line, Cell Culture, Irradiation, and Transfection

The human GBM cell lines U87MG was acquired from the Korea Cell Line Bank (KCLB, Seoul, Korea), and the phenotypes of these cell lines have been authenticated by the KCLB. All cells were free of mycoplasma contamination and were authenticated by short tandem repeat profiling within the past 12 months. The cells were grown in MEM medium supplemented with 10% FBS, penicillin-streptomycin (10,000 U/mL) at 37 °C in a humidified atmosphere of 95% air and 5% CO_2_. Patient-derived BCL20-HP02 glioblastoma stem cell lines were obtained from patients undergoing resection in accordance with a protocol approved by Haeundae Paik Hospital (Inje University, Busan, Korea). The patient-derived glioblastoma stem cell lines were cultured in DMEM/F-12 supplemented with B27, EGF (20 ng/mL), bFGF (20 ng/mL), penicillin-streptomycin (10,000 U/mL) at 37 °C in a humidified atmosphere of 95% air and 5% CO_2_. For delivery of X-ray by employing an x-ray generator M-150WE (Softex, Tokyo, Japan). For transient transfection, the mixture of Lipofectamine^TM^ RNAiMAX (Invitrogen, Carlsbad, CA, USA) and siRNA oligonucleotide (10 nmol/L) targeting DYRK3 was incubated 30 min for formation of liposome and applied to the U87MG cells and BCL20-HP02 cells for 36 h. After exchanging with fresh media, the cells were treated the recombinant human EGF protein, which could increase mTORC1 activity above basal levels, for 45 min and harvested for further experiments. The study protocol using the patient-derived glioblastoma stem cells was approved by the Institutional Review Board of Haeundae Paik Hospital (Inje University, Busan, Korea) on 30 June 2017 (IRB No. HPIRB-2017-06-007-004).

### 4.3. Animal Care Protocol and Orthotopic Xenograft Mouse Model

This method was performed as previously described [22]. Six-wk-old male BALB/c athymic nude mice (Orient Bio, Seongnam, Korea) were used for the in vivo experiments. The animal protocols were approved by the Institutional Animal Care and Use Committee of Pusan National University (Busan, Korea), and experiments were performed under provisions of the National Institutes of Health’s Guide for the Care and Use of Laboratory Animals. The mice were maintained in animal care facilities in a temperature-regulated room (23 ± 1 °C) under a 12 h light/dark cycle and were fed water and standard mouse chow ad libitum. The U87MG cells were harvested and suspended at a density of 1 × 10^5^ cells/μL in serum-free media. Then, 5 × 10^5^ cells were stereotactically injected into the brain of mice (*n* = 5 for each group, weight: 18 ± 2 g). 18 days after the injection date, tumor xenografts were irradiated with 2 Gy daily for 5 days at a dose rate of 600 MU/min using a TrueBeam STx (Varian Medical Systems, Palo Alto, CA, USA). The radiation was delivered by using an 8 mm-diameter collimator. At the end of the treatment period, the animals were euthanized and brain tumor samples were harvested. The survival rates of orthotopically xenografted mice were increased by radiation treatment (Mantel–Cox test: *p* = 0.0273).

### 4.4. Total RNA Isolation and qRT-PCR

Expression levels of mRNAs was analyzed by real-time qRT–PCR, as previously described [51]. Total RNA was extracted from U87MG cells or xenograft tumor tissues with Trizol reagent (Invitrogen, Carlsbad, CA, USA) according to the manufacturer’s instructions, after which 1 μg of total RNA was used for cDNA synthesis and Real-time qRT-PCR was performed using an Applied Biosystems StepOne Real-Time PCR System (Applied Biosystems, Foster City, CA, USA). It was performed for 40 cycles of 95 °C for 15 s and 60 °C for 1 min followed by thermal denaturation. The sequences of used primers are listed below (Table 1). 

### 4.5. Immunohistochemistry (IHC)

IHC was performed as previously described [51]. The mouse brain tissues were fixed in formalin, dehydrated, and embedded in paraffin blocks, which were then sectioned at 4 μm. Sections were incubated in 3% hydrogen peroxide/methanol and then in 0.25% pepsin (S3002; Dako, Carpinteria, CA, USA). The samples were blocked in blocking solution (X0909, 10062794; Dako), incubated at 4 °C overnight with specific primary antibodies diluted in antibody diluent (1:200, S3022, 10064048; Dako), washed with TBST, and incubated with polymer-HRP-conjugated secondary antibody (K4001, 10063530 for anti-mouse IgG; K4003, 10061639 for anti-rabbit IgG; Dako). A 3,3′-diaminobenzidine substrate chromogen system (K3468, 10063768; Dako) was used to detect antibody binding. The sections were inspected under an Olympus IX71 inverted microscope (Olympus Optical, Tokyo, Japan).

### 4.6. Western Blot Analysis

After the desired treatments, whole cell lysates were prepared using ProEXTM CETi Lysis Buffer (with protease and phosphatase inhibitors, TransLab, Daejeon, Korea) and the concentrations of protein were determined using a BioRad protein assay kit (BioRad Laboratories, Hercules, CA, USA). The protein samples were subjected to SDS-PAGE, transferred to the 8% nitrocellulose membrane, and then blocked with 5% bovine serum albumin in TBST (10 mM Tris, 100 mM NaCl, and 0.1% Tween 20) for 40 min at room temperature. Next, the membranes were probed with specific primary antibodies at 4 °C overnight and subsequently probed with peroxidase-conjugated secondary antibody (Santa Cruz Biotechnology, Santa Cruz, CA, USA). The membranes were visualized using an ECL detection system (Roche Applied Science, Indianapolis, IN, USA) with iBright chemi-doc fl000 from Thermo Fisher Scientific (Thermo Fisher Scientific, Cleveland, OH, USA). For each analysis, at least three biological replicates were performed.

### 4.7. MitoTracker^TM^ Green FM and MitoSOX^TM^ Red Staining

Cells seeded in confocal dish (101350; SPL Life Sciences, Pocheon, Korea) were incubated with MitoTracker Green^FM^ probe at a final concentration of 250 nM in HBSS at 37 °C (5% CO_2_/95% air) for 45 min and MitoSOX probe at a final concentration of 5 μM in HBSS at 37 °C (5% CO_2_/95% air) for 10 min. Next, cells were rinsed with pre-warmed HBSS three times and added fresh media in the confocal dish. Finally, cells were examined under an Olympus IX71 inverted microscope (Olympus Optical Co., Ltd., Tokyo, Japan).

### 4.8. Transmission Electron Microscopy (TEM)

The material was pre-fixed with 2.5% glutaraldehyde in PBS (pH 7.2) at 4 °C and was post-fixed with 1% osmium tetroxide in PBS. The material was dehydrated with a series of the graded ethyl alcohol and embedded in epoxy resin (Epon 812 mixture). Thick sections were sectioned at 1 μm and stained with 1% toluidine blue for light microscope. Thin sections were sectioned at 50~60 μm and prepared by using an ultramicrotome (EM UC7, Leica, Wetzlar, Germany) and were double stained with uranyl acetate and lead citrate. Thin sections were analyzed with a transmission electron microscope (JEM-1200EXⅡ, JEOL, Tokyo, Japan). This experiment was performed at Pusan National University Hospital (Busan, Korea).

### 4.9. Seahorse Analysis

OCR and ECAR were determined using Seahorse XFp Analyzer (Agilent Technologies, Santa Clara, CA, USA) [22]. Cells were plated on Seahorse XFp plates at a concentration of 1.5 × 10^3^ cells/well for 24 h at 37 °C in a humidified atmosphere of 95% air and 5% CO_2_. The cells were washed and incubated with Seahorse XF Base Medium (Agilent Technologies, Santa Clara, CA, USA) at 37 °C for 1 h in a non-CO_2_ incubator. For the OCR assay, injection port A on the sensor cartridge was loaded with oligomycin (complex V inhibitor, final concentration 2.5 μM), port B was loaded with FCCP (uncoupler of oxidative phosphorylation, final concentration 1.5 μM), and port C was loaded with rotenone/antimycin A (inhibitors of complex I and complex III, final concentration 1.0 μM each). For the ECAR assay, injection port A on the sensor cartridge was loaded with glucose (final concentration 10 μM), port B was loaded with oligomycin (final concentration 2.2 μM), and port C was loaded with 2-D-glucose (competitive inhibitor of glucose metabolism, final concentration 100 μM). After the Seahorse XFp flux analysis, cells were lysed to calculate the protein concentration. The results were normalized to protein abundance in corresponding wells. For each analysis, a minimum of three wells were utilized per condition to calculate OCR and ECAR, and at least three biological replicates were performed.

### 4.10. Migration and Invasion Assay

Effects of knockdown of DYRK3 and irradiation on cell migration/invasion capacity were investigated by transwell cell migration/invasion assay, as previously described [52]. Cells (5 × 10^4^ in serum-free MEM medium) were seeded into the upper chambers of a 24-well Transwell chamber (Corning, Corning, NY, USA) fitted with 5 μm pore-size insert and treated with DYRK3 siRNA and/or radiation, for 24 h. Next, the lower chamber was changed into MEM medium containing 10% FBS. After 12 h, the upper membrane surface was wiped with a cotton swab to remove cells that had not migrated into the lower side of the membrane. Then the upper chambers were fixed with 70% EtOH, stained with 0.05% crystal violet, and photographed by microscope AXIO from Carl Zeiss (Carl Zeiss, Oberkochen, Germany).

### 4.11. Three-Dimensional (3D) Culture

3D culture was performed as previously described [53]. Briefly, growth factor-reduced matrigel (BD Biosciences, Franklin Lakes, NJ, USA) was thawed overnight at 4 °C and cells were cultured on 8-well chambered glass slides (Ibidi USA, Fitchburg, WI, USA) with matrigel. The glass slides were incubated at 37 °C for 1 h and the cells were resuspended as a single-cell suspension of 25,000 cells/mL in the media. Next, 200 μL of the cell suspension was mixed with 200 μL of media containing 4% matrigel and dispensed into each well of the glass slides. The cells were then incubated and allowed to attach at 37 °C in a 5% CO_2_ atmosphere for 3 days. For immunofluorescence staining, the cells were fixed with 4% paraformaldehyde for 20 min, then permeabilized in 0.1% Triton X-100 for 10 min. The cells were subsequently washed three times with PBS, after which they were blocked in IF buffer (PBS with 0.1% BSA, 0.2% Triton X-100, and 0.05% Tween 20) containing 10% FBS at 37 °C for 1 h. Next, the samples were stained with primary anti-α-tubulin antibody overnight at 4 °C and then washed three times with IF buffer. Following incubation with DyLight 488-conjugated secondary antibodies (Thermo Fisher Scientific, Cleveland, OH, USA) and counter-staining and mounted with Fluoroshield Mounting Medium with DAPI (ab104139; Abcam, Cambridge, MA, USA). Fluorescent images were visualized with the Olympus IX71 fluorescence microscope (Olympus Optical Co., Ltd., Tokyo, Japan).

### 4.12. Database Analysis and Statistical Analysis

GlioVis tool was utilized for analysis of the differential expressions of DYRK3 according to subtype and prognosis of glioma patients. All numerical data are presented as the means ± standard error of the mean from at least three independent and randomized experiments. Data were analyzed using *t*-tests for quantifications. The Prism 5 software (GraphPad Software, San Diego, CA, USA) was used for all statistical analyses. A *p*-value < 0.05 was considered to be statistically significant. 

## Figures and Tables

**Table 1:** Primers used for qRT-PCR.

Target Gene	Primer Sequences
Human GAPDH	FW 5′- ATGACATCAAGAAGGTGGTG -3′ RV 5′- CATACCAGGAAATGAGCTTG -3′
Human DYRK3	FW 5′- CCCTCTGCCCGCTTGAC-3′ RV 5′- CCCGTTTCCCTGACACCT T -3′

## Data Availability

Data is contained within the article.

## Abbreviations

DRP1	Dynamin related protein 1
DYRK3	Dual specificity tyrosine-phosphorylation-regulated kinase 3
GBM	Glioblastoma multiforme
mTORC1	Mammalian target of rapamycin complex 1
OXPHOS	Oxidative phosphorylation
PRAS40	Proline-rich AKT1 substrate 1
ROS	Reactive oxygen species
